# The Role of Oxidative Stress-Mediated Inflammation in the Development of T2DM-Induced Diabetic Nephropathy: Possible Preventive Action of Tannins and Other Oligomeric Polyphenols

**DOI:** 10.3390/molecules27249035

**Published:** 2022-12-18

**Authors:** Gohar Sahakyan, Anne Vejux, Naira Sahakyan

**Affiliations:** 1 Research Institute of Biology, Yerevan State University, 1 A. Manoogian Str., Yerevan 0025, Armenia; 2 Team “Biochemistry of the Peroxisome, Inflammation and Lipid Metabolism”, University Bourgogne Franche-Comté, UFR Sciences Vie Terre et Environnement, 21000 Dijon, France; 3 Research Institute of Biology, Department of Biochemistry, Microbiology & Biotechnology, Yerevan State University, 1 A. Manoogian Str., Yerevan 0025, Armenia

**Keywords:** cytokines, insulin resistance, diabetic nephropathy, inflammation, ROS, tannins, polyphenols

## Abstract

Diabetic nephropathy is manifested in more than 10% of people with diabetes. It is a common cause of kidney failure and end-stage kidney disease. Understanding of mechanisms underlying the initiation and development of diabetes-induced kidney injuries will allow for the development of more effective methods of prevention and treatment of the disease. Diabetic nephropathy is a wide-ranging complication of diabetes, and it is necessary to discuss the “weight” of pro-inflammatory pathways and molecules in the progress of renal injuries during the development of the disease. A large spectrum of pro-inflammatory molecules and pathways participate in different stages of the pathophysiological progression of diabetic nephropathy, including pro-inflammatory cytokines, chemokines, their receptors, adhesion molecules, and transcription factors. On the other hand, it is known that one of the consequences of hyperglycemia-induced ROS generation is the up-regulation of pro-inflammatory cascades, which, in turn, activate the transcription of genes encoding cytokines-chemokines, growth factors, and extracellular matrix proteins. It is a proven fact that a variety of plant secondary metabolites, such as tannins, flavonoids, and other polyphenols, demonstrate significant anti-diabetic, redox-modulating properties and effectively modulate the inflammatory response. Thus, this review is discussing the possible role of plant phenols in the prevention and treatment of diabetic nephropathy.

## 1. Introduction

The development of type 2 diabetes mellitus (T2DM) is a result of insulin resistance (IR) in organisms, which is associated with an inability of insulin to stimulate glucose uptake by target cells and to reduce the blood glucose concentration. As a compensatory response of the body, insulin secretion by the pancreas increases, and hyperinsulinemia is developed. The progression of IR induces the inability of target cells to react to insulin and results in the development of T2DM. The main physiological causes of IR are nutritional overload and accumulation of certain lipids and their metabolites in cells, low physical activity, chronic inflammation, and stress of various natures, including oxidative [1].

A direct correlation between hyperglycemia-induced oxidative stress, inflammation, and the development and progression of T2DM has been proven. Thus, hyperglycemia-induced oxidative stress increases the levels of pro-inflammatory proteins with infiltrated macrophages secreting inflammatory cytokines. As a result, local and systemic inflammation is developed, which in its turn enhances the production of ROS, and consequently, the progression of T2DM goes deeper.

Following Stanley Schwartz, hyperglycemia and T2DM can have three main causes: systemic inflammations, pathological changes of the intestinal micro-flora, and disorders of amylin synthesis [2].

Concerning the latter, the parallel between Alzheimer’s disease and T2DM was drawn [3]. They have shown the key role of amyloids in the development in both cases. So, in the case of the Alzheimer’s disease, amyloid-β (Aβ) brings the loss of neurons, while in the case of the T2DM, amylin (human pancreatic islet amyloid polypeptide (hIAPP)) damages insulin-produced β-cells. The investigations showed that this demonstrates the formation of Aβ-hIAPP heterocomplex aggregates as a result of interaction between these two amyloidogenic proteins [4], and the accumulation of these complexes in the brain and pancreas is associated with cell dysfunction and death. Following these findings, it can be stated that mitochondrial dysfunction observed in both diseases is also a result of the accumulation of amyloids. Authors point out that a quarter of amyloid-damaged proteins are mitochondrial. Amyloids inhibit the IV complex in mitochondria (cytochrome C oxidase (CytOx), which is the last enzyme in the respiratory chain) [5,6]. This results in mitochondrial dysfunction with severe consequences both in Alzheimer’s disease and DM.

DeFronzo pointed out eight more factors, the so-called “ominous octet”, that contributed to the pathophysiology of type 2 diabetes: IR of hepatic cells; IR of other target cells; decreased insulin secretion due to dysfunction of β-cells; disorders of incretin effect; hyperfunction of α-cells and, as a result, an increase of glucagon synthesis; increased lipolysis due to activation of lipases in adipocytes; increased glucose reabsorption in kidneys; and neurotransmitter dysfunction in the central neural system [7].

Diabetic nephropathy (DN) is a wide-ranged complication of diabetes, which is in direct connection with the IR, increasing the level of a large spectrum of pro-inflammatory molecules (pro-inflammatory cytokines, chemokines, and their receptors, adhesion molecules, and transcription factors) and pathways participating in different stages of the pathophysiological progression. On the other hand, it is known that one of the consequences of hyperglycemia-induced ROS generation is the up-regulation of pro-inflammatory cascades, which, in turn, activate the transcription of genes encoding cytokines-chemokines, growth factors, and extracellular matrix proteins.

Evidence-based medicine accepts the treatment of individuals with DN by the control of high blood sugar and hypertension, and therapy with angiotensin-converting enzyme (ACE) inhibitors or angiotensin receptor blockers (ARBs), which can slow or halt the progression of diabetic renal disease in early stages. In addition to the ACE inhibitor and/or ARBs, sodium-glucose co-transporter-2 (SGLT2) inhibitors and nonsteroidal selective mineralocorticoid receptor antagonists (MRAs) should be used [8].

Nowadays, the use of plant-origin bioactive metabolites in medicine is highly actual due to their significant redox-modulating and anti-inflammatory properties in different systems. In particular, plant polyphenols can be used as agents for decreasing blood glucose levels, improving insulin resistance, protecting islets, decreasing oxidative stress, inhibiting inflammation, and Maillard reaction and advanced glycation end products (AGEs) formation [9,10,11,12,13].

In this context, there is a huge interest in understanding the potential benefit of these substrates in the prevention and treatment of DN.

The goal of this review is the analysis and summary of the literature data of the last five years concerning the mechanisms of induction, development, and progression of DN, as well as the evaluation of the potential usage of plant polyphenols as the prevention and treatment agents for diabetes-induced kidney failure.

## 2. Hyperglycemia and Diabetic Nephropathy

It is known that hyperglycemia induces the increase of the osmotic pressure of biological fluids due to a decrease in the glucose absorption rate of cells [14]. As a result, diuresis develops, which means loss of water and salts from kidneys, water exhaustion, and deficits of ions. On the other hand, the intensity of non-enzymatic glycosylation of proteins and lipids increases in these conditions [15].

One of the most typical diabetic complications is diabetic nephropathy (DN), which usually decreases the life quality, brings invalidation, and is the largest single cause of end-stage renal disease [16,17].

DN is characterized by the development of sclerosis of the renal glomeruli, leading to impaired renal function, primarily the filtration function of the kidneys, and the development of chronic renal failure [18].

Following the modern classification [19] DN has three stages of development: microalbuminuria (MAU); proteinuria (PU); and chronic kidney disease (CKD).

MAU is recognized as an early predictor of nephropathy [20]. In this stage, the albumin excretion is 30 and 300 mg/day; in patients with T2DM, high blood pressure can be observed, and it is important to point out that it will be possible to recover kidney functions if treatment starts on time [21].

PU is known as a visible or real stage of DN. In this stage, albumin in urea is above 300 mg/day, kidney glomerular filtration rate (GFR) decreases, and stable high blood pressure is observed. Treatment is based on angiotensin change enzyme (ACE) inhibition or angiotensin receptor blocking [22].

CKD is a terminal stage of DN. This stage is typically characterized by a high level of blood creatinine and urea; GFR is less than 60 mL/min, and hypertension progresses. When GFR becomes less than 15 mL/min, kidney therapy is necessary (hemodialysis, dialysis, and even kidney transplantation) [17,23].

An understanding of the mechanisms of DN is a necessity and there are a lot of studies on this path [24,25]. A large amount of literature data are available regarding the polyethiological nature of DN. The most attention is given to the genetic, metabolic, and vascular factors [26,27].

There is an accepted fact that in the basis of pathological mechanisms of DN development, three factors are underlying: hyperglycemia and dyslipidemia; intraglomerular hypertension; and arterial hypertonia, where oxidative stress plays a crucial role [27]. 

Hyperglycemia is an initial metabolic “button” of the DN. On the one hand, the high-level glucose leads to the non-enzymatic glycosylation of glomerular proteins and lipids and damages glomerular vesicles. On the other hand, glucose increases vascular permeability due to its direct toxic effect on kidney tissue [27]. As a result, the tone of the adductor blood vessel decreases, and it expands, while the efferent vessel saves its tension due to the effect of angiotensin II [28]. These changes bring renal hypertension and, as a sequel, glomerulosclerosis develops. Arterial hypertonia, which is typical of T2DM, contributes to the progression of glomerulosclerosis as well [2].

## 3. T2DM and Inflammation

Despite diabetes not being thought to be an immune disease, there are many studies pointing to the role of inflammation in T2DM [29]. In this context, it is important to note the influence of TNFα, IL1, and IL6 in the pathophysiological mechanisms of IR [30]. Animal experiments have shown increased levels of TNFα in adipose tissue in obesity; its neutralization restored glucose uptake by peripheral tissues [31]. These results were also confirmed for humans, where the TNFα level correlates with insulin resistance (IR) and decreases with weight loss [32]. Finally, a mechanism has been demonstrated whereby inflammatory cytokines can interrupt signaling from the insulin receptor internally [33]. All these data formed the basis for the creation of the concept of the relationship between inflammation and IR.

**Figure 1:**
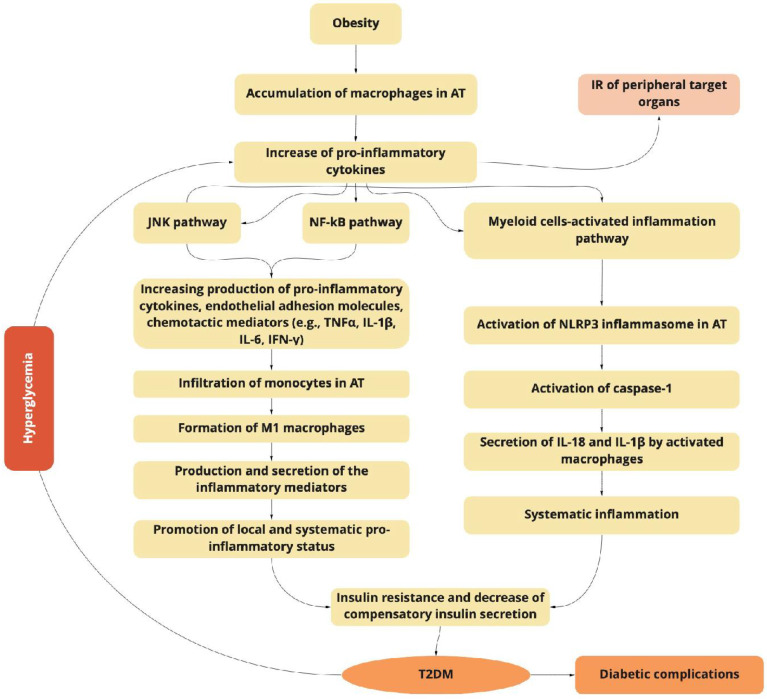
The obesity-related inflammation as a predictor of type 2 diabetes mellitus (T2DM) induction and progression.

T2DM is an obesity-related metabolic syndrome with the sustained activation of the NLRP3 inflammasome, which is a critical component of the innate immune system mediating caspase-1 activation and the secretion of proinflammatory cytokines IL-1β/IL-18. The activation of the NLRP3 inflammasome is linked with inflammatory disorders such are Alzheimer’s disease, diabetes, and atherosclerosis. The NLRP3 inflammasome is activated by different factors and diverse molecular and cellular events such as ionic flux, production of ROS, mitochondrial dysfunction, and lysosomal damage [30,34]. It is important to point out that T2DM is accompanied by oxidative stress-induced ROS production, which could result in NF-κB activation and the transcription of NLRP3 and, as a result, the activation of pro-IL-1β and pro-IL-18 [34,35] (Figure 1).

The obesity-related accumulation of macrophages in adipose tissue (AT) activates the JNK and NF-κB signaling pathways, increasing the production of pro-inflammatory cytokines, endothelial adhesion molecules, and chemotactic mediators, which contribute to the infiltration of monocytes, and the formation of pro-inflammatory M1 macrophages. Macrophage-secreted diverse inflammatory mediators promote the local and systemic pro-inflammatory state and induce insulin resistance of targeted tissues. The NF-κB inflammatory pathway results in the increased expression of several NF-κB target genes (e.g., IL-6, TNFα, IFN-γ, and IL-1β), exacerbating the IR progression. Myeloid cells activate the inflammasome pathway connected with the macrophages and other innate immune cells. The last ones initiate inflammatory responses by detecting pathogen- or danger-associated molecular patterns by pattern-recognition receptors such as NLRP3, which plays a key role in the obesity-specific chronic inflammation and progression of IR. The activation of caspase-1 mediates the secretion of IL-1β and IL-18 by macrophages. The increasing production of IL-1β in pancreatic islets and insulin-sensitive tissues is associated with T2DM. IL-18 enhances the maturation of T- and NK-cells, and increases the production of diverse pro-inflammatory cytokines, exacerbating obesity-induced systemic inflammation.

Activation of the NLRP3 inflammasome in adipose-tissue-infiltrating macrophages brings metabolic inflammation, which in its turn aggravates the inflammation in insulin-sensitive tissues [36]. During β-cell failure, activation of NLRP3 could be released via alternative mechanisms. Initially, due to hyperglycemia, the β-cell-derived mitochondrial ROS is produced, which leads to the dissociation of thioredoxin-interacting protein from thioredoxin and then the activation of NLRP3 [37]. Furthermore, continuous hyperinsulinemia brings the accumulation of a large amount of IAPP around the islet cells, which, in its turn, specifically, activates the NLRP3 inflammasome [38]. Animal studies using mice with beta-cell-specific overexpression of IAPP revealed a strong induction of IL-1β in pancreatic macrophages [39].

## 4. Inflammation and DN

**Figure 2:**
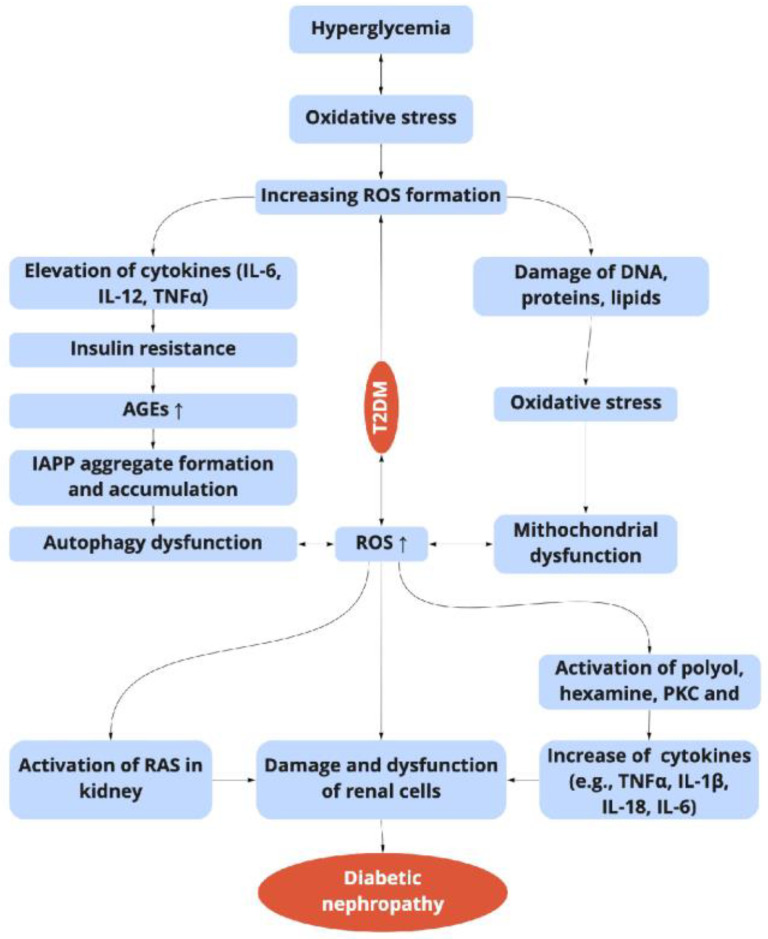
Relationship between the hyperglycemia-induced oxidative stress, inflammation, and diabetic nephropathy in T2DM. ROS: reactive oxygen species; TNF-α: tumor necrosis factor-α; NF-κB: nuclear transcription factor-κB; AGEs: advanced glycation end products; PKC: protein kinase; IAPP: islet amyloid polypeptide; RAS: renin-angiotensin system.

A wide range of changes including hemodynamic and metabolic disorders, the upregulation of the renin-angiotensin system (RAS), oxidative stress, and fibrosis are the main characteristics of DN [40]. All these alterations together bring the increase of systemic and intraglomerular pressure, and the development of diverse symptoms associated with the development of kidney failure, such as glomerular hypertrophy, and decreasing glomerular filtration [23]. Recent advances indicate that kidney complications in DM are not only a result of alterations in hemodynamic and metabolic factors, but a complex and multifactorial process (Figure 1 and Figure 2) [26]. 

Following the last studies, inflammation plays a key role in the pathophysiological mechanisms of DN [41]. In this context, it is necessary to discuss the “weight” of pro-inflammatory pathways and molecules in the progress of renal damage during the development of the disease. A large spectrum of pro-inflammatory molecules and pathways participate in the pathophysiological processes of diabetic nephropathy, including pro-inflammatory cytokines, chemokines, their receptors, adhesion molecules, and transcription factors [42]. The increase of pro-inflammation cytokines in the blood of patients has been noted, and a direct correlation has been found between DN progression, albuminuria, and the increase in the concentration of the pointed cytokines [43,44].

Numerous inflammatory parameters foretold the initiation and progression of DN [45]. Inflammatory cytokines play a binary role. They regulate the immune response and play key roles as basic promoting elements of injury. The elevated concentrations of these elements in T2DM patients trigger microvascular complications, particularly the development of nephropathy [46]. Both the circulating pro-inflammatory cytokines and those synthesized and secreted by inflammatory cells in the kidney tissue are increased in patients with DN, and, simultaneously, are in direct correlation with urinary albumin excretion (UAE) levels and clinical markers of glomerular and tubulointerstitial damage [47].

The pathophysiological changes observed at various levels of DN contribute to the development and progression of kidney damage [48].

Inflammatory mechanisms are crucial in the DN pathophysiology and explain how metabolic and hemodynamic disorders in DM patients translate to structural and functional kidney injuries.

Thus, IL-18 is a proinflammatory cytokine synthesized by renal tubular cells and by infiltrated monocytes, macrophages, and T-cells as well [49]. These cytokine levels are elevated in the serum and urine of patients with DN. Significant and direct correlations are observed between IL-18 and UAE levels, and consequently, the evolution of albuminuria [50]. The level of IL-18 could be used as an early marker of renal dysfunction in T2DM patients. Additionally, the levels of IL18 in serum correlate with the level of β2-microglobulin in urine, which is a marker of tubular dysfunction [50]. It is important to note that IL18 modulates the synthesis of IL-1, TNF-α, and interferon γ (IFN-γ), which in its turn activates chemokine receptors in mesangial cells. Moreover, IL8 enhances the expression of intercellular adhesion molecule 1 (ICAM-1) [50] and facilities endothelial cell apoptosis [37]. In addition to infiltrating cells, kidney tubular cells of patients with DN express raised levels of IL-18 as well, which is connected with the activation of the mitogen-activated protein kinase (MAPK) pathways by transforming growth factor (TGF) β [51].

Serum and urinary levels of TNF-α elevate in patients with DN in parallel with the progression of renal injuries, which may point to a relationship with the development and progression of renal deficiency [52]. It was shown that the increasing level of TNF-α has a cytotoxic effect on the renal tissue, while the inhibition of TNF-α improves markers of glomerular and tubulointerstitial injuries in DN patients [53]. Some investigations are stating that the increasing levels of TNF-α in kidney glomeruli and tubules are directly and independently associated with the UAE [45,54].

In the pathogenesis of DN, IL-1 is also involved [55]. This cytokine triggers the synthesis of prostaglandin E and the release of phospholipase A2. The latter play a critical role in the progression of intraglomerular hemodynamic abnormalities. Due to the influence of IL-1, the permeability of vascular endothelial cells increases as well [50].

In animal DN models, the activation of IL-1 expression is revealed in many types of renal cells [56,57]. The relationship between IL1 activity and the expression of intercellular adhesion molecule 1 (ICAM-1), as well as the vascular cell adhesion molecule-1 (VCAM-1), and the endothelial-leukocyte adhesion molecule-1 (ELAM-1), have been shown [58]. Rat kidney mesangial cells produce prostaglandin E2 after incubation with recombinant IL-1 in response to angiotensin II, which might result in the appearance of abnormalities in intraglomerular hemodynamics [50]. IL-1 is associated with the secretion of hyaluronan in the proximal tubule, which is related to the progression of hypercellularity [59].

IL-6 is another cytokine that is involved in DN progression due to its pleiotropic effects [60]. The increase of IL-6 enhances proliferation of the extracellular matrix and influences vascular permeability which in its turn brings to DN. IL-6 participates in facilitating the neutrophil infiltration of the tubule-interstitium, acts on extracellular matrix dynamics, and contributes to overall kidney hypertrophy, thickening the renal glomeruli and podocyte hypertrophy, correlating with albuminuria [61].

## 5. DN and Oxidative Stress

As already mentioned, hyperglycemia is an accepted key factor for the development of diabetic microvascular complications such as nephropathy [62,63]. Previously, it was shown that elevated glucose concentrations may influence the proliferation of renal cells (glomerular mesangial cells and proximal tubular epithelial cells) in vitro by the alteration of cytokine generation [56]. On this basis, clear protocols were built for developing cellular models of diabetic nephropathy and studying their metabolism [64].

It is well-recognized that hyperglycemia results in the raised generation of superoxide (O_2_^−^) from diverse sources including mitochondria, NADPH oxidase, and uncoupled nitric oxide synthase (NOS) [62,65]. In its turn, O^2−^ could react with nitric oxide, which brings to the removal or attenuation of NO protective effects on the vascular system and generation of peroxynitrite [66].

It is important to note that nitric oxide is essential for vasorelaxation [67]. It penetrates vascular smooth muscle cells and activates soluble guanylyl cyclase (sGC), forming cyclic guanosine monophosphate (cGMP)-elicited vasorelaxation. This highlights the importance of the NO/sGC/cGMP pathway in the kidneys [68]. On the other hand, as mentioned above, peroxynitrite radical (ONOO-) production depends on the production rates of NO and O_2_^−^ in biological systems [69]. Peroxynitrite is a strong oxidant, which is able to promote one- and two-electron oxidations by direct reactions with bio-molecular targets. In addition, peroxynitrite can evolve the secondary radicals via its fast reaction with CO_2_ or through proton-catalyzed homolysis. The modification of biomolecules by nitration or oxidation can bring failures in bioenergetics which underlies the physiopathological conditions such as neurodegenerative diseases, ischemia-reperfusion, diabetes, endotoxic shock, and aging. In addition, peroxynitrite radical can also damage proteins and DNA with subsequent activation of poly-ADP ribose polymerase [70]. It leads to the poly-ADP ribosylation of different proteins including glyceraldehyde-3-phosphate dehydrogenase. As a result, the increasing availability of glycolytic intermediates induces their diversion into other pathways such are the polyol pathway, the hexosamine pathway, the protein kinase C (PKC) pathway, and the advanced glycation end (AGE) products pathway, which are closely associated with the development of the microvascular and macrovascular complications in DM [71,72]. Thus, NO can play a bipolar role in kidneys being a vasorelaxant or serving as a substrate for the synthesis of peroxynitrite, depending on the presence of ROS.

In its turn, the activation of the PKC pathway increases NF-κB and subsequent pro-inflammatory gene expression [42]; increases NADPH oxidase activity and triggers the generation of reactive oxygen species (ROS) [73]; and activates plasminogen activator inhibitor-1 (PAI-1)-induced reduction in fibrinolysis. These bring fibrosis and end-stage renal disease (Figure 2).

Thus, it is clear that one of the consequences of hyperglycemia-induced ROS generation is the upregulation of pro-inflammatory cascades which, in turn, activate the transcription of genes encoding cytokines/chemokines, growth factors, and extracellular matrix proteins.

## 6. The Perspective of Tannins as Potential Anti-Inflammatory and Antioxidant Agents

The above-mentioned studies prove the pivotal role of inflammation in the initiation and progress of DN in T2DM [25] (Figure 1). Strong and complex interconnections exist between oxidative stress and inflammation processes [41]. Alterations of the redox state play a crucial role in many cellular processes, including in the activation/dysfunction of innate immune cells [42,73]. On the other hand, it is proven that plant-origin phenolic substances, despite some complications in bioavailability, possess significant redox-modulating properties in different models and effectively modulate the inflammatory response [74,75,76,77]. Along these lines, polyphenols are among the most investigated compounds [78,79].

Tannins are a group of naturally occurring high molecular weight nitrogen-free polyphenols which are present in almost all investigated plant species. According to the chemical structure, tannins are classified into four main categories [80], which can be sorted into hydrolyzable and condensed (proanthocyanidins) groups. The solubility in water is a key factor for expressing the biological activities of these substances. Hydrolyzable tannins are esters of gallic acid or ellagic acid, with a sugar core (glucose), and are hydrolyzed by acids or enzymes into monomeric products. More than 500 hydrolyzable tannins have been described until now [80]. They consist of polyphenol nuclei with molecular weights ranging from 500 to 3000 Da. The condensed tannins are oligomeric or polymeric flavonoids composed of flavone-3-ols, including catechin, epicatechin, gallocatechin, and epigallocatechin. Their molecular weights vary from 1000 to 20,000 Da [81]. They can be depolymerized only with strong oxidation and are hardly degraded by anaerobic enzymes [82].

Animal and human in vivo studies have demonstrated that the bioavailability of polyphenols can vary depending on the experimental system and their chemical structure [83,84]. The phenomenon of the bioavailability of tannins is of interest. Thus, Afsana et al. [85] have found that approximately 85% of ingested tannins disappeared from the rat intestine. They have suggested that the main part of the ingested tannins was hydrolyzed in the large intestine and absorbed as gallic acid or was further degraded. Nakamura et al. have shown that more than 60% of these substances remained in an intact form after oral ingestion, but that some were hydrolyzed to gallic acid by bacterial tannases in the intestine and further metabolized to 4-0-methyl gallic acid, pyrogallol, and resorcinol [86]. Nowadays, there are a lot of studies concerning the bioavailability of tannins because of their perspective usage in medicine, agriculture, and the food industry [81,83,87,88,89].

Numerous papers indicated the beneficial health effects of tannins [83,87,89]. Thus, condensed tannins are effective against diverse types of allergies such as asthma, hypersensitive pneumonitis, and allergic rhinitis. Tannins possess various biological applications such as anti-inflammatory, anti-cancer, anti-allergic, anthelmintic, and antiviral [87,89]. They are used as anti-hemorrhagic, antidiarrheal agents since ancient times. In addition, tannins also act as precipitating agents and have beneficial effects on vascular health [90,91].

Some reports have shown that various tannins such as gallic acid, ellagic acid, catechin, epicatechin, and procyanidins extracted from medicinal plants participate in controlling the progression of diabetes and related complications due to their action on molecular pathways and the main targets involved in progression [75,92]. These findings are used as a pharmacophore for developing new preparations with enhanced therapeutic benefits in the treatment of diabetic complications. It was shown that tannins reduce the risk of diabetes by enhancing glucose uptake and thus lowering the levels of blood sugar [88,92].

Tannins were systematically studied in the past few decades for their anti-inflammatory and antioxidant effects [75,93,94].

Following the literature reviewed, the antioxidant function of plant tannins mainly depends on their chemical structure rather than the extraction source [83,93]. Some researchers suggest that tannins show antioxidant activity because of the high-degree of hydroxylation of aromatic rings due to their high molecular weight [95]. The ability to bind free radicals depends on the number of hydroxyl groups: the more hydroxyl groups in tannins, the more easily they can be oxidized [96].

According to Castaldo et al. [95], the antioxidant property of tannins prevents cholesterol oxidation, which is a precursor of plaque formation in vessels, thus preventing the body from cardiovascular diseases. Some authors have shown that tannins can protect against acute doxorubicin-induced cardiotoxicity by inhibiting inflammation, oxidative stress, and apoptosis. Furthermore, tannins suppress lipid oxidation by scavenging different radicals [97,98], and decrease the arsenic trioxide-induced nephrotoxicity by simultaneous inhibition of nuclear factor-kappa (NF-κB) and activation of the nuclear factor-erythroid-2-related factor 2 (Nrf2) pathways [99].

The anti-inflammatory and wound healing potential of tannins extracted from seedling leaf tissue and callus culture extracts of *Achyranthes aspera* L. and *Ocimum basilicum* L. has been studied using four rabbit models, i.e., excision, incision, dead space, and burn. They have shown that the anti-inflammatory activity of callus cultures of leaf explants was comparable with the standard drug Indomethacin [99].

Wu et al. found that plant tannins manifest anti-inflammatory effects by inhibiting NO and prostaglandin-E2 (PGE2) [92]. Liu et al. demonstrated that grape seed procyanidin extract can reduce obesity-induced inflammation by mediating the expression of cytokines [100]. Another study revealed that the tannin fraction extracted from black raspberry seeds has anti-inflammatory activity due to the reducing nitric oxide (NO) induced by lipopolysaccharide (LPS) in RAW 264.7 cells [101]. Moghrovyan et al. [102] demonstrated other mechanisms of anti-inflammatory activities of plant-origin phenolic substances.

**Figure 3:**
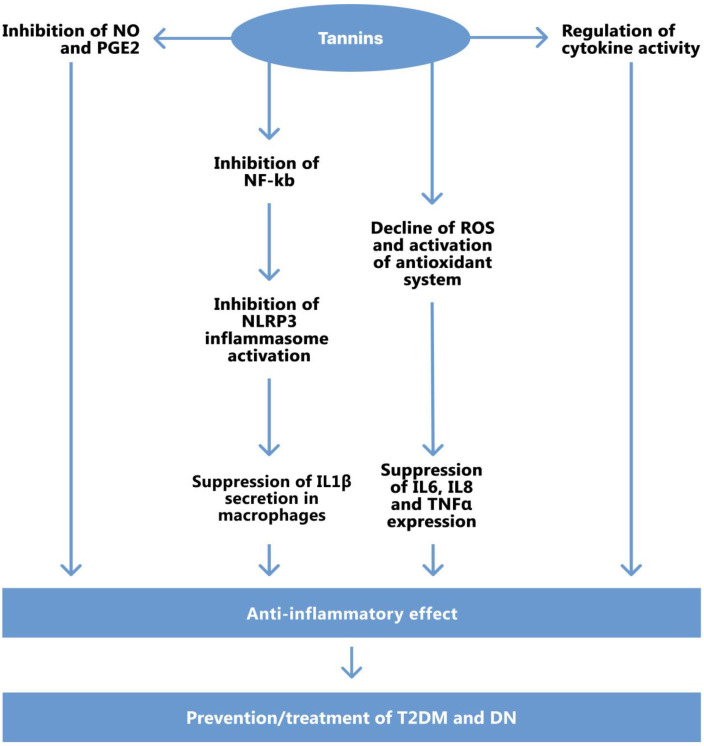
Possible pathways of the anti-inflammatory effect of tannins.

It is suggested that the anti-inflammatory properties of tannins may be released by regulating cytokine expression, reducing the production of inflammatory substances, and enhancing the formation of complexes with other molecules [89]. Tannic acid declines the levels of reactive oxygen species (ROS), malondialdehyde (MDA), and, at the same time, inclines activities of superoxide dismutase (SOD), catalase (CAT), glutathione (GSH). It also suppresses expressions of IL-6, IL-8, and TNF-α. It was reported that tannins inhibited NLRP3 inflammasome activation by blocking NF-κB signaling to suppress IL-1β secretion in macrophages (Figure 3). The authors suggest that the data obtained provide evidence that tannic acid may be a potent inhibitor for NLRP3-driven diseases [92].

Tannins could modulate cytokine activity directly. On the other hand, this effect could be realized by the regulation of the antioxidant system and the inhibition of NO, PGE2, NF-kB, and the activation of NLRP3 inflammasome.

Taking into account the literature data on the role of oxidative stress and inflammation in the initiation and progression of T2DM and T2DM-induced DN, we can assume that tannins can be applicable as a means for the prevention and treatment of these diseases.

### Possible Role of Plant Polyphenols in the Prevention and Treatment of DN

As it was stated previously, even in the norm, the renal tissue is susceptible to hypoxia, which, in turn, induces and aggravates oxidative stress, and in turn, exacerbates the renal hypoxia. Oxidative stress is enhanced in diabetic kidney disease and contributes to the progression of renal injury. The imbalance between the pro-oxidant and antioxidant systems exist in DN with an overproduction of ROS due to chronic hyperglycemia and diminished expression of antioxidant enzymes, which play a crucial role in the pathogenesis of diabetic nephropathy arousing metabolic and cellular disturbances (lipid peroxidation, protein oxidation, and DNA damage), and stimulating the inflammatory response [103].

In addition to the main endogenous antioxidants, such as superoxide dismutase (SOD), catalase, glutathione peroxidase (GSH-Px), haem oxygenase-1 (HO-1), and the thioredoxin, glutathione (GSH), there are also several exogenous antioxidants, such as vitamins and plant polyphenols with possible positive influence in the regulation of redox balance in the organism. Numerous data from the literature have demonstrated the positive influence of plant secondary metabolites such are polyphenols in the treatment of DN (Figure 3) [12,104,105,106,107,108,109,110,111,112]. Ma et al. have shown that baicalin, a bioactive flavonoid from the root of the medicinal plant *Scutellaria baicalensis*, can treat DN by alleviating oxidative stress and inflammation by the activation of the Nrf2-mediated antioxidant signaling pathway, and the inhibition of the MAPK-mediated inflammatory signaling pathway [105].

The beneficial effects of quercetin [106], resveratrol [107], cordycepin [108], different flavonoids [109], allicin [110], ursolic acid [111], and epigallocatechin-3-gallate [112] are stated during the treatment of kidney damages. For instance, in a rat model of adenine-induced chronic kidney disease, treatment with quercetin improved renal function by reduction of oxidative stress factors, serum levels of fibroblast growth factor-23 (FGF23), and kidney inflammation. In case of resveratrol and also other mentioned plant-origin compounds, the renal function can be improved by suppressing inflammation and oxidative stress in different rodent models via different mechanisms.

Despite the facts indicating the key role of oxidative stress in DN initiation and progression, the usage of plant antioxidants has not become a standard yet for the treatment of patients with DN because of the lack of information concerning the clear mechanisms of the action of these substances. As a result, further investigation is still needed to fill this gap.

## 7. Conclusions

Summarizing the above-mentioned, it can be stated that T2DM has become one of the most challenging public health problems in the world due to its increasing prevalence and mortality rates, which demand more effective therapeutic agents, especially for the complications of DM. Literature data discussed above have shown that plant polyphenols have cardio-protective, neuroprotective, anti-oxidative, and anti-inflammatory effects. Plant tannins directly benefit DM by decreasing blood glucose levels, improving insulin resistance, inhibiting inflammation, decreasing oxidative stress, and inhibiting advanced glycation end-product formation. They could also benefit DM indirectly by retarding and improving a series of DM complications, such as DN.

Thus, it may be possible to suggest that plant polyphenols might be potential adjuvant agents for the future prevention and treatment of DM and DN. However, comprehensive studies of its effects and mechanisms are still needed.

## Data Availability

Not applicable.
